# Expression, Cellular and Subcellular Localisation of Kv4.2 and Kv4.3 Channels in the Rodent Hippocampus

**DOI:** 10.3390/ijms20020246

**Published:** 2019-01-09

**Authors:** Rocío Alfaro-Ruíz, Carolina Aguado, Alejandro Martín-Belmonte, Ana Esther Moreno-Martínez, Rafael Luján

**Affiliations:** Synaptic Structure Laboratory, Instituto de Investigación en Discapacidades Neurológicas (IDINE), Dept. Ciencias Médicas, Facultad de Medicina, Universidad Castilla-La Mancha, Campus Biosanitario, C/Almansa 14, 02008 Albacete, Spain; Rocio.Alfaro@uclm.es (R.A.-R.); Carolina.Aguado@uclm.es (C.A.); Alejandro.Martin@uclm.es (A.M.-B.); anaesther.moreno@uclm.es (A.E.M.-M.)

**Keywords:** hippocampus, potassium channel, electron microscopy, immunohistohemistry, histoblot

## Abstract

The Kv4 family of voltage-gated K^+^ channels underlie the fast transient (A-type) outward K^+^ current. Although A-type currents are critical to determine somato-dendritic integration in central neurons, relatively little is known about the precise subcellular localisation of the underlying channels in hippocampal circuits. Using histoblot and immunoelectron microscopic techniques, we investigated the expression, regional distribution and subcellular localisation of Kv4.2 and Kv4.3 in the adult brain, as well as the ontogeny of their expression during postnatal development. Histoblot demonstrated that Kv4.2 and Kv4.3 proteins were widely expressed in the brain, with mostly non-overlapping patterns. During development, levels of Kv4.2 and Kv4.3 increased with age but showed marked region- and developmental stage-specific differences. Immunoelectron microscopy showed that labelling for Kv4.2 and Kv4.3 was differentially present in somato-dendritic domains of hippocampal principal cells and interneurons, including the synaptic specialisation. Quantitative analyses indicated that most immunoparticles for Kv4.2 and Kv4.3 were associated with the plasma membrane in dendritic spines and shafts, and that the two channels showed very similar distribution patterns in spines of principal cells and along the surface of granule cells. Our data shed new light on the subcellular localisation of Kv4 channels and provide evidence for their non-uniform distribution over the plasma membrane of hippocampal neurons.

## 1. Introduction

Principal cells and interneurons of the hippocampus are fundamental for information processing in this brain region [1]. The main hippocampal trisynaptic circuit that relays cortical input to granule cells in the dentate gyrus on to pyramidal neurons in the *cornu Ammonis* 3 (CA3) region and then to pyramidal neurons in the CA1 region is important for spatial navigation, memory consolidation and decision making [1]. These and other functions of hippocampal neurons depend on their synchronous activity, which is controlled by organised excitatory and inhibitory inputs to dendritic spines and shafts and by intrinsic conductances that cause them to fire. Intrinsic activity depends on electrical signals largely determined by ion channels operating along the neuronal plasma membrane. The dysfunction of ion channels known to regulate hippocampal neurophysiology is linked to pathologies including schizophrenia, epilepsy and Alzheimer’s disease [2,3].

Voltage-gated potassium (Kv) channels are one type of ion channel controlling the excitability and function of hippocampal neurons [4]. Among Kv channels, Kv4 channels underlie somato-dendritic rapidly inactivating (A-type) currents, which are important determinants of dendritic excitability [5,6,7]. Electrophysiological, pharmacological and immunohistochemical data suggest that a major component of the somato-dendritic A-type Kv current is formed by the *Shal* or Kv4 family [4,6,7]. These channels are formed by four Kv4 α subunits (Kv4.1, Kv4.2 and Kv4.3), but only Kv4.2 and Kv4.3 are predominant in the brain. Native neuronal Kv4 channels associate with two types of auxiliary subunits: Membrane-spanning dipeptidyl aminopeptidase-like proteins (DPPs) and cytoplasmic Kv channel interacting proteins (KChIPs) [8,9]. KChIPs are Ca^2+^ binding proteins that bind to the cytoplasmic N-terminal domain of Kv4 [8,10] and also interact with Alzheimer’s disease-associated presenilin-2 [11,12]. Four subtypes of KChIP proteins (KChIP1–KChIP4) have been cloned [8,10,11,12,13]. In the brain, KChIPs regulate biophysical, biochemical and cell biological properties of Kv4 channels.

In situ hybridisation and immunohistological studies have established that Kv4.2 and Kv4.3 channels are differentially and widely expressed in the brain [14,15,16,17]. The hippocampus exemplifies a brain region where the Kv4.2 and Kv4.3 channels are expressed at high levels [14,15,16,17]. At the cellular level, Kv4.2 mRNA and protein has been reported to be present in hippocampal pyramidal cells [14,15,16,17], supported by electrophysiological studies showing that Kv4.2 channels underlie the somato-dendritic A-current in pyramidal neurons in the CA1 region of the hippocampus [18]. In contrast, Kv4.3 mRNA and protein was uniquely observed in hippocampal interneurons in the CA1 region, and also in principal cells in the CA3 region and dentate gyrus [15,17], suggesting the presence of heterotetrameric Kv4.2/Kv4.3 channels in these cells. Little information is available, however, regarding how these proteins become organised in different subcellular compartments. Therefore, to unravel the subcellular localisation of Kv4.2 and Kv4.3 channels in hippocampal cells we used immunoelectron microscopy approaches combined with quantitative analyses. Our results demonstrate that these subunits have a characteristic subcellular compartment-specific distribution.

## 2. Results

### 2.1. Differential Regional Expression of Kv4.2 and Kv4.3 Channels in the Adult Brain

To determine the regional expression of Kv4.2 and Kv4.3 channels in the brain, we used subunit-specific antibodies in conventional immunohistoblotting. This technique is a reliable way to analyse the brain expression of different proteins without compromising the integrity of antibody-binding sites by tissue fixation that is commonly required for immunohistochemistry [19,20]. In addition, the histoblot reflects the spatial pattern in which proteins are arranged within a brain section and affords accurate quantitative analyses in the adult and developing brain.

**Figure 1:**
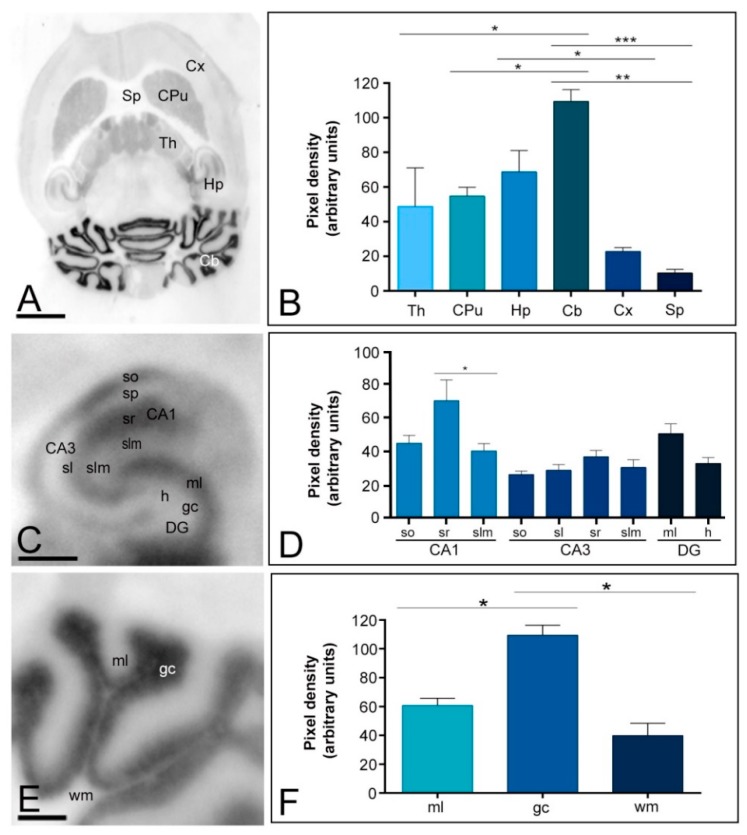
Regional distribution of the Kv4.2 channel in the adult mouse brain. (**A**,**B**) The distribution of the Kv4.2 protein was visualised in histoblots of horizontal brain sections at P60 using an affinity-purified anti-Kv4.2 antibody. The expression of Kv4.2 in different brain regions was determined by densitometric analysis of the scanned histoblots. The strongest expression was detected in the cerebellum (Cb) and hippocampus (Hp), with moderate expression in the caudate putamen (CPu) and thalamus (Th). Weak expression level was detected in the cortex (Cx) and septum (Sp); (**C**,**D**) In the hippocampus, very strong Kv4.2 expression was detected in the *strata oriens* (so) and *radiatum* (sr) of the CA1 region and the molecular layer (ml) of the dentate gyrus (DG); (**C**,**D**) Moderate staining was observed in all dendritic layers of CA3, in the *stratum lacunosum-moleculare* (slm) of the CA1 region and the *hilus* of the dentate gyrus. Very weak Kv4.2 staining was observed in the *stratum pyramidale* of the CA1 and CA3 regions and in the granule cell layer of the dentate gyrusso, *stratum oriens*; sr, *stratum radiatum*; DG, dentate gyrus; h, *hilus*.; (**E**,**F**) In the cerebellum, the strongest expression level was detected in the granule cell layer (gc), with weak expression in the molecular layer (ml) and very weak in the white matter (wm). Error bars indicate SEM; * *p* < 0.05; ** *p* < 0.01; *** *p* < 0.001. Scale bars: A, 1 mm; C and E, 0.5 mm.

In the adult brain (P60), the overall expression of Kv4.2 and Kv4.3 channels revealed marked regional-specific differences. Immunoreactivity for Kv4.2 was strongest in the cerebellum and hippocampus (Figure 1A,B). Moderate staining was detected in the caudate putamen and thalamus, faint in the neocortex and midbrain nuclei, and weak in the septum (Figure 1A,B). The hippocampus and cerebellum, the two regions showing the highest Kv4.2 expression, were further examined in region- and layer-specific analyses (Figure 1C–F). In the hippocampus, immunolabelling for Kv4.2 was strongest in the *strata oriens* and *radiatum* of the CA1 region and in the molecular layer of the dentate gyrus (Figure 1C,D). Moderate staining was observed in all dendritic layers of CA3, in the *stratum lacunosum-moleculare* of the CA1 region and the *hilus* of the dentate gyrus (Figure 1C,D). Very weak Kv4.2 staining was observed in the *stratum pyramidale* of the CA1 and CA3 regions and in the granule cell layer of the dentate gyrus (Figure 1C). In the cerebellum, immunoreactivity for Kv4.2 was low in the molecular layer, highest in the granule cell and very low in the white matter (Figure 1E,F).

**Figure 2:**
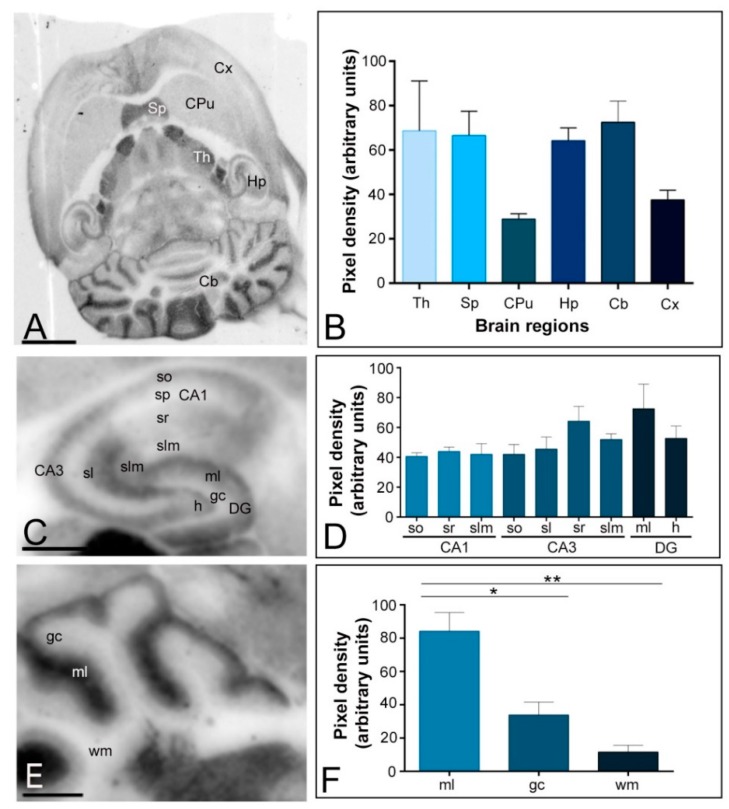
Regional distribution of the Kv4.3 channel in the adult mouse brain. (**A**,**B**) The distribution of the Kv4.3 protein was visualised in histoblots of horizontal brain sections at P60 using an affinity-purified anti-Kv4.3 antibody. Kv4.3 expression in different brain regions was determined by densitometric analysis of the scanned histoblots. The strongest expression was detected in the cerebellum (Cb), thalamus (Th) septum (Sp) and hippocampus (Hp), with weaker expression in the cortex (Cx) and caudate putamen (CPu); (**C**,**D**) In the hippocampus, strong Kv4.3 expression was detected in the *stratum radiatum (sr)* of the CA3 region and in the molecular layer (ml) of the dentate gyrus (DG), moderate labelling was observed in all dendritic layers of the CA1 region, the *strata oriens* (so)*. Lucidum* (sl) *and lacunosum-moleculare* (slm) of the CA3 region, as well as in the *hilus* (h) of the dentate gyrus. Very weak Kv4.3 staining was evident in the *stratum pyramidale* of the CA1 and CA3 regions and in the granule cell layer of the dentate gyrus; (**E**,**F**) In the cerebellum, the strongest expression level was detected in the molecular layer (ml), weak expression in the granule cell layer (gc) and very weak in the white matter (wm). Error bars indicate SEM; * *p* < 0.05; ** *p* < 0.01. Scale bars: **A**, 1 mm; **C** and **E**, 0.5 mm.

Immunoreactivity for Kv4.3 in the adult brain (P60) was strongest in the cerebellum, thalamus, septum and hippocampus (Figure 2A,B). Moderate staining was detected in midbrain nuclei, including the inferior and superior colliculus, and brainstem nuclei (Figure 2A). Faint staining was observed in the neocortex and caudate putamen (Figure 2A,B). The hippocampus and cerebellum were further examined in region- and layer-specific analyses (Figure 2C–F). In the hippocampus, immunoreactivity for Kv4.3 was very strong in the *stratum radiatum* of the CA3 region and in the molecular layer of the dentate gyrus (Figure 2C,D). Moderate labelling was observed in all dendritic layers of the CA1 region, the *strata oriens. lucidum and lacunosum-moleculare* of the CA3 region, as well as in the *hilus* of the dentate gyrus (Figure 2C,D). Very weak Kv4.3 staining was evident in the *stratum pyramidale* of the CA1 and CA3 regions and in the granule cell layer of the dentate gyrus (Figure 2C). In the cerebellum, immunolabelling for Kv4.3 was significantly stronger in the molecular layer than in the granule cell layer, in which moderate to weak labelling was consistently detected, and it was very weak in the white matter (Figure 2E,F). Altogether, these data revealed that both Kv4.2 and Kv4.3 are widely expressed in the adult brain but show differences in a brain region-specific manner.

### 2.2. Expression Patterns of Kv4.2 and Kv4.3 Channels during Postnatal Development

**Figure 3:**
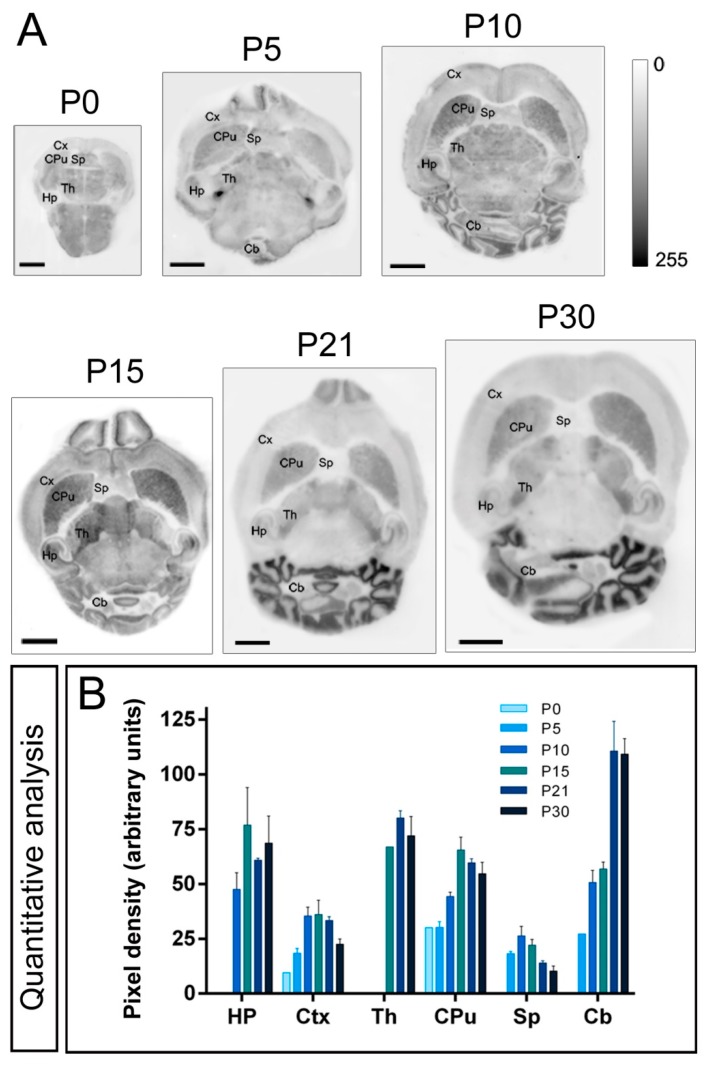
Developmental and regional distribution of the Kv4.2 channel in the mouse brain. (**A**) Kv4.2 protein distribution was visualised on histoblots of brain horizontal sections at various stages of postnatal development using an affinity-purified anti-Kv4.2 antibody. Kv4.2 was expressed in the brain since the day of birth (P0), and at all stages the strongest expression was detected in the cerebellum (Cb), caudate putamen (CPu), hippocampus (Hp) and thalamus (Th), with the lowest intensity in the cortex (Cx) and septum (Sp); (**B**) The histoblots were scanned and densitometric measurements from four independent experiments were averaged to compare the protein densities for each developmental time point. The analysis revealed a differential Kv4.2 expression in a developmental stage- and region-specific manner. Kv4.2 expression was detected at P0, increased progressively to reach a peak at P10, P15 or P21 depending on the brain region, and then decreasing at P30. Error bars indicate SEM. Scale bars, 2 mm.

To determine the developmental profile of Kv4.2 and Kv4.3 protein expression during postnatal development, histoblot analyses were performed in the brain. The Kv4.2 and Kv4.3 proteins were expressed in the developing brain from the day of birth (P0), showing significant differences in a region- and channel-specific manner. For instance, in the hippocampus, weak Kv4.2 expression was detected at P0 and then increased progressively to reach a peak at P15, followed by a decrease at P21 and P30 (Figure 3A,B). This developmental expression profile was obtained from the region showing the highest level, the *stratum radiatum* of the CA1 area. Expression patterns in other brains regions differed to that described in the hippocampus. Thus, in the cortex weak Kv4.2 expression was detected at P0, increased progressively to reach a peak at P10, P15 and 21, and decreased at P30 (Figure 3A,B). In the caudate putamen, weak expression was detected at P0 and P5, increased progressively to reach a peak at P15, and decreased towards P30 (Figure 3A,B). In the septum, weak Kv4.2 expression was detected at P5, increased progressively to reach a peak at P10, decreasing progressively towards P30 (Figure 3A,B). In the cerebellum, weak expression for Kv4.2 was detected at P5, increased progressively to reach a plateau at P10 and P15, and then increased again to reach a peak at both P21 and P30 (Figure 3A,B). This developmental expression profile was obtained from the region showing the highest level, the granule cell layer of the cerebellar cortex.

**Figure 4:**
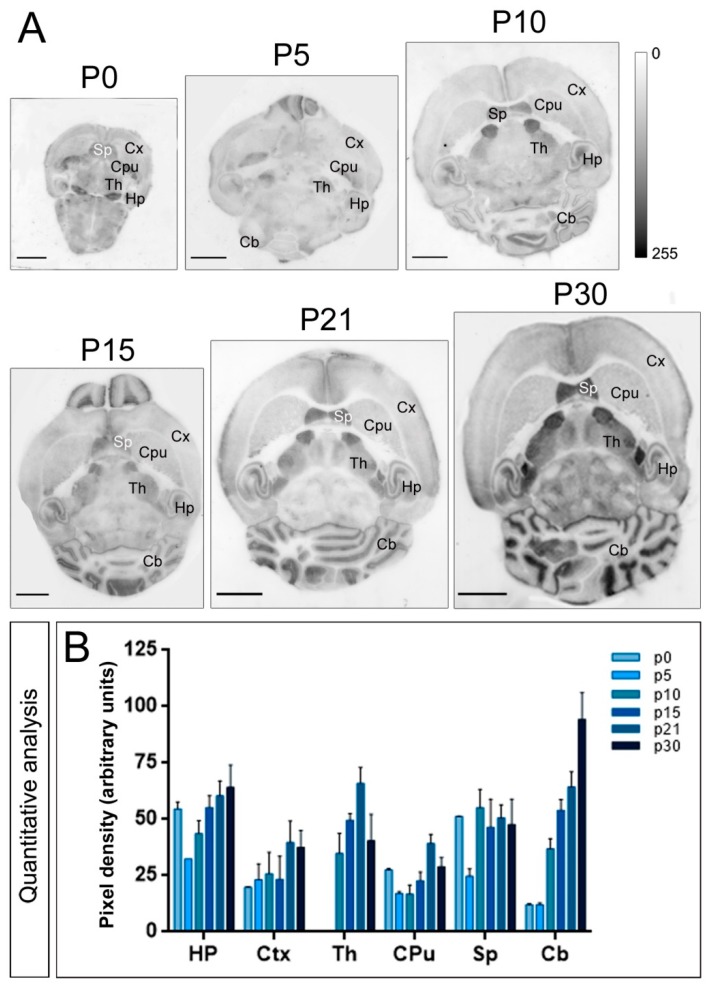
*Developmental and regional distribution of the Kv4.3 channel in the mouse brain*. (**A**) Kv4.3 protein distribution was visualised on histoblots of brain horizontal sections at various stages of postnatal development using an affinity-purified anti-Kv4.3 antibody. Kv4.3 was expressed in the brain since the day of birth (P0), and at all stages the strongest expression was detected in the cerebellum (Cb), thalamus (Th), hippocampus (Hp) and septum (Sp), with the lowest intensity in the caudate putamen (CPu) and cortex (Cx); (**B**) The histoblots were scanned and densitometric measurements from four independent experiments were averaged to compare the protein densities for each developmental time point. The analysis revealed a differential Kv4.3 expression in a region- and developmental-dependent manner. Kv4.3 expression was detected at P0, thought at some time points was not possible to quantify. Then, expression increased progressively to reach a peak at P10, P15 or P21 depending on the brain region, and then decreasing at P30 in cortex, thalamus, caudate putamen and septum, or increasing until P30 in hippocampus and cerebellum. Error bars indicate SEM. Scale bars, 2 mm.

The developmental expression of Kv4.3 in the brain was different from that described for Kv4.2 above. In the hippocampus, quantifying the *stratum radiatum* of the CA3 area that showed the highest level, weak Kv4.3 expression was detected and high at P0, decreased at P5 and then increased progressively to reach a peak at P30 (Figure 4A,B). A similar expression developmental pattern was detected in the cerebellum (Figure 4A,B) but was different in other brain regions. Thus, in the cortex, weak Kv4.3 expression was detected from P0 to P15, increased to reach a peak at P21, and decreased slightly at P30 (Figure 4A,B). In the caudate putamen, weak Kv4.3 expression was detected and high at P0, decreased at P5, increased progressively to reach a peak at P21, decreasing at P30 (Figure 4A,B). In the septum, the expression of Kv4.3 at P0 was high, decreased at P5, and then increased to reach a plateau from P10 until P30 (Figure 4A,B). Altogether, these data show that Kv4.2 and Kv4.3 have a unique and differential developmental expression pattern.

### 2.3. Distribution of Immunoreactivity for Kv4.2 and Kv4.3 as Detected by Light Microscopy

To determine the regional and cellular distribution of Kv4.2 and Kv4.3 in the adult hippocampus, light microscopy immunohistochemical analyses were performed. Immunoreactivity for Kv4.2 or Kv4.3 in the hippocampus showed distinct distribution patterns with partial overlap. Immunoreactivity for Kv4.2 and Kv4.3 was widely distributed in all subfields of the hippocampus, showing differential labelling patterns. Thus, although both ion channel subtypes were present throughout the hippocampus, in some subfields they were localised to distinct sets of neurons, whereas in others they were both expressed by the same cell types.

#### 2.3.1. Immunoreactivity for Kv4.2. 

**Figure 5:**
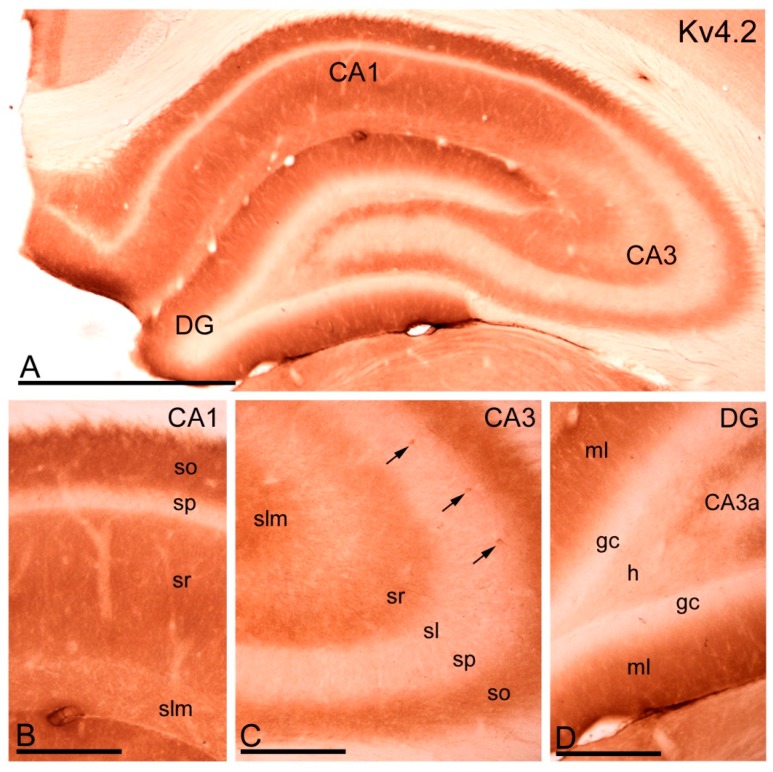
Distribution of immunoreactivity for Kv4.2 in the hippocampus. (**A**) At the light microscopic level, immunoreactivity for Kv4.2 was widely distributed in the hippocampus but its intensity varied consistently; (**B**) In the CA1 region, immunolabelling for Kv4.2 was generally moderate-to-strong, with the *strata oriens* (so) and *radiatum* (sr) showing the highest and the *stratum lacunosum-moleculare* (slm) showing weak immunoreactivity. The weakest immunolabelling for Kv4.2 was observed in the *stratum pyramidale* (sp); (**C**) In the CA3 region, moderate immunolabelling for Kv4.2 was observed in the *strata oriens* (so), *radiatum* (sr) and *stratum lacunosum-moleculare* (slm), weak in the *stratum lucidum* and very weak in the *stratum pyramidale* (sp). Kv4.2 immunolabelling was also seen outlining somata and dendrites of scattered interneurons (arrows); (**D**) In the dentate gyrus (DG), immunolabelling was strong in the molecular layer (ml), weak in the *hilus* (h) and the weakest in the granule cell layer (gc). Scale bars: **A**, 500 µm; **B**–**D**, 150 µm.

**Figure 6:**
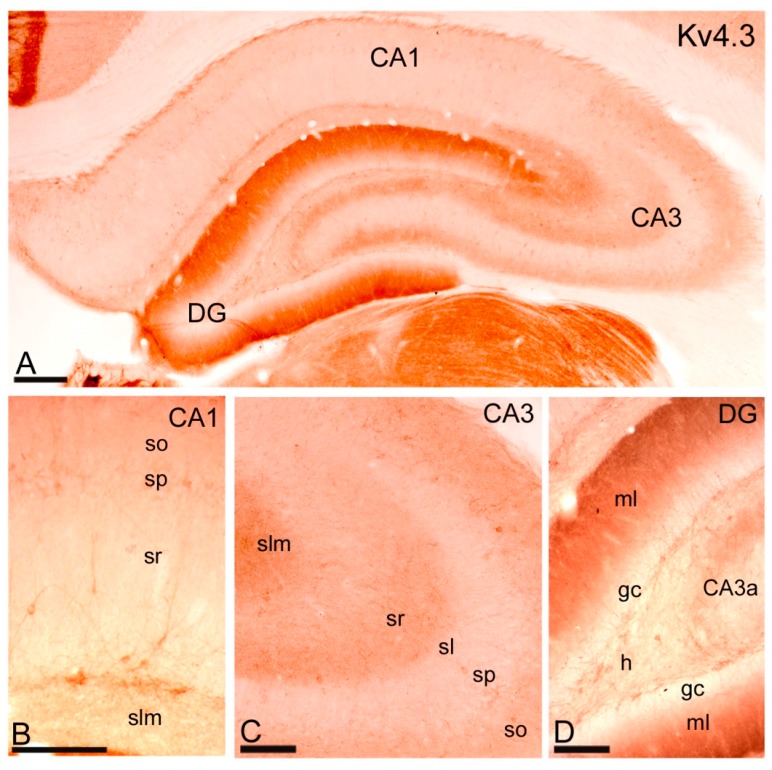
Distribution of immunoreactivity for Kv4.3 in the hippocampus. (**A**) Immunoreactivity for Kv4.3 was widely distributed in the hippocampus although cellular distribution varied in a subfield-dependent manner; (**B**) In the CA1 region, immunoreactivity for Kv4.3 was observed exclusively in the soma and dendrites of interneurons distributed throughout the *strata oriens* (so), *pyramidale* (sp), *radiatum* (sr) and *lacunosum-moleculare* (slm); (**C**) In the CA3 region, immunolabelling for Kv4.3 was detected along the soma and dendrites of interneurons scattered throughout the *strata oriens* (so), *pyramidale* (sp), *radiatum* (sr) and *lacunosum-moleculare* (slm). In addition, strong neuropilar staining that indicate expression in pyramidal cells was observed in in *strata oriens* (so), *radiatum* (sr) and *lacunosum-moleculare* (slm) with a weaker labelling in the *stratum lucidum* (sl), but very weak in the *stratum pyramidale* (sp); (**D**) In the dentate gyrus (DG), immunolabelling for Kv4.3 was strong in the neuropil of the molecular layer (mL) and weak in the *hilus* (h), and also strong in somata and dendrites of scattered interneurons. The granule cell layer of the dentate gyrus showed very weak immunolabelling for Kv4.3. Scale bars: **A**, 200 µm; **B**–**D**, 100 µm.

All hippocampal subfields were immunolabelled for Kv4.2, although the intensity of immunoreaction varied consistently (Figure 5). The CA1 region was the most strongly immunoreactive in the hippocampus, the *strata oriens* and *radiatum* being especially intensely labelled, as well as the molecular layer of the dentate gyrus (Figure 5A,B,D). The *stratum lacunosum-moleculare* in the CA1 region and all dendritic subfields of the CA3 region were labelled less intensely (Figure 5A–C). The *stratum lucidum* of the CA3 region and the *hilus* of the dentate gyrus were only weakly immunopositive (Figure 5C,D). In addition to this neuropilar staining described in all dendritic layers, Kv4.2 immunoreactivity could also be seen outlining cell bodies and dendrites of scattered interneurons (Figure 5C), but many fewer cell bodies were visualised than with antibodies to Kv4.3 (Figure 6). The *stratum pyramidale* in the CA1 and CA3 regions, as well as the granule cell layer in the dentate gyrus, were the least Kv4.2 immunoreactive areas of the hippocampus (Figure 5A–D).

#### 2.3.2. Immunoreactivity for Kv4.3. 

Labelling for Kv4.3 was observed both in the soma and dendrites of interneurons and in the neuropil (Figure 6A). In the CA1 region, immunolabelling for Kv4.3 was only detected in the soma and dendrites of interneurons distributed throughout all CA1 subfields, including the *stratum pyramidale* (Figure 6A,B). In the CA3 region, immunolabelling for Kv4.3 was strong in the neuropil in *strata oriens*, *radiatum* and *lacunosum-moleculare* with a weaker labelling in the *stratum lucidum* (Figure 6A,C). In addition to neuropilar staining that indicate expression in pyramidal cells, immunolabelling for Kv4.3 was clearly visible on the soma and dendrites of interneurons scattered throughout the different dendritic layers of the CA3 region (Figure 6A,C). In the dentate gyrus, immunolabelling for Kv4.3 was strong in the neuropil of the molecular layer and weak in the *hilus*, and also strong in somata and dendrites of scattered interneurons (Figure 6A,D). In the pyramidal cell layer of the CA1 and CA3 regions, and the granule cell layer of the dentate gyrus, very weak immunolabelling for Kv4.3 was detected (Figure 6A–D).

### 2.4. Distribution of Immunoreactivity for Kv4.2 and Kv4.3 as Detected by Electron Microscopy

**Figure 7:**
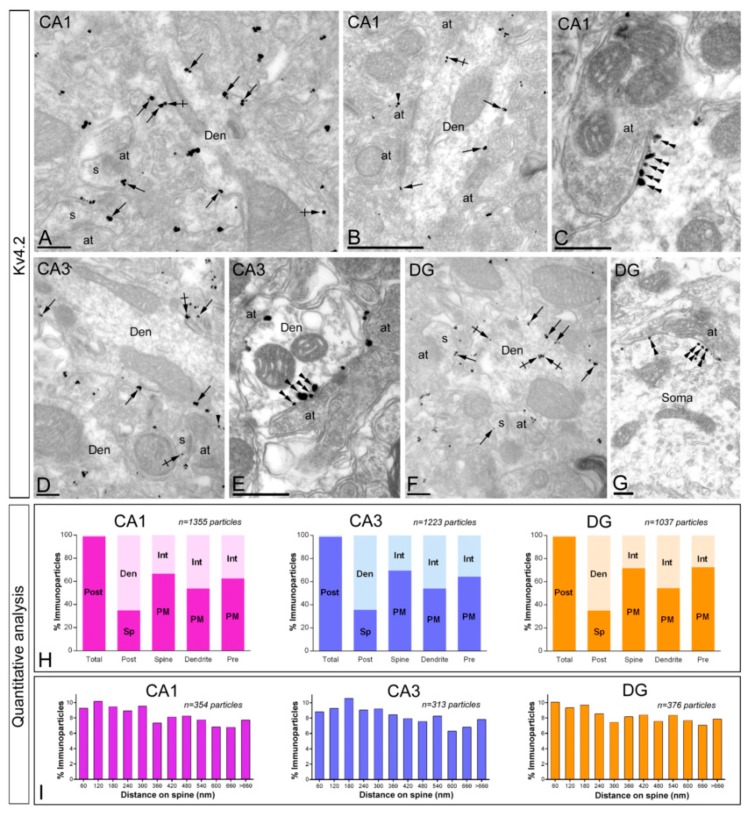
Subcellular localisation of Kv4.2 in the adult hippocampus. Electron micrographs showing immunoparticles for Kv4.2 in the hippocampus, as detected using the pre-embedding immunogold technique at P60. (**A**–**D**) Immunoreactivity for Kv4.2 was detected in similar neuronal compartments in the CA1 (panels **A**, **B** and **C**) and CA3 (panels **D** and **E**) regions and dentate gyrus (DG, panels **F** and **G**). Kv4.2 immunoparticles were abundant along the extrasynaptic plasma membrane (arrows) of dendritic spines (s) contacted by axon terminals (at) and dendritic shafts (Den) of pyramidal cells and granule cells. Immunoparticles were also observed at intracellular sites (crossed arrows) in dendritic spines (s) and shafts (Den). Very few immunoparticles for Kv4.2 were also localised to the extrasynaptic plasma membrane (arrowheads) of axon terminals (at) establishing asymmetrical synapses with spines (s). Kv4.2 immunoparticles (double arrowheads) were detected along the main body of the postsynaptic membrane specialisation of GABAergic synapses in the CA1 (panel **C**), CA3 (panel **E**) and DG (panel **G**); (**H**) Compartmentalisation of Kv4.2 in CA1 pyramidal cells, CA3 pyramidal cells and DG granule cells. Bar graphs showing the percentage of immunoparticles for Kv4.2 at post- and presynaptic compartments, and along the plasma membrane and intracellular sites in dendritic spines, dendritic shafts and axon terminals. A total of 1355 immunoparticles in the CA1, 1223 in the CA3 and 1037 in the DG were analysed. Most immunoparticles were postsynaptic, both along the plasma membrane and at cytoplasmic sites. Postsynaptically, immunoparticles were detected in dendritic spines and in dendritic shafts; (**I**) Histogram showing the distribution of immunoreactive Kv4.2 in relation to glutamate release sites in dendritic spines of CA1 and CA3 pyramidal cells, and DG granule cells. Data are expressed as the proportion of immunoparticles at a given distance from the edge of the synaptic specialisation. These data show that Kv4.2 immunoparticles were distributed similarly in spines in CA1, CA3 and DG and in the proximity of asymmetrical synapses on dendritic spines. Scale bars: **A**,**D**,**F**,**G**, 200 nm; **B**,**C**,**E**, 500 nm.

**Figure 8:**
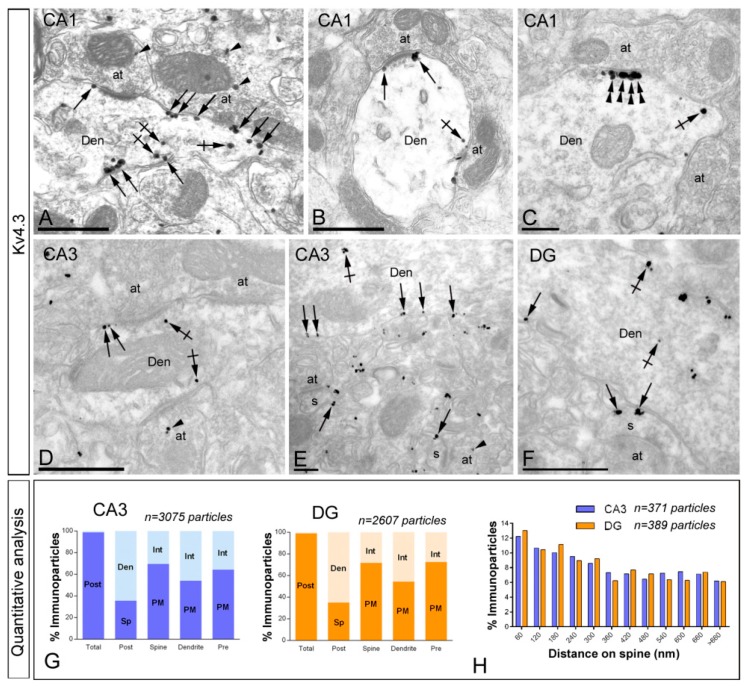
Subcellular localisation of Kv4.3 in the adult hippocampus. Electron micrographs showing immunoparticles for Kv4.3 in the hippocampus, as detected using the pre-embedding immunogold technique at P60. (**A**–**C**) In the CA1 region, immunoparticles for Kv4.3 were detected along the extrasynaptic plasma membrane (arrows) of dendritic dendritic shafts (Den) of interneurons contacted by both excitatory and inhibitory axon terminals (at) in the *strata oriens* and *radiatum* but less frequent in the *stratum lacunosum-moleculare*. In addition, immunoparticles were observed at cytoplasmic sites (crossed arrows) associated with intracellular membranes. Immunoparticles are present at the edge of the synaptic membrane specialisations (double arrowheads), at synapses between the dendrite (Den) of an interneuron in the *stratum oriens* and axon terminals (at); (**D**–**F**) In the CA3 region and DG, immunoreactivity for Kv4.3 was detected along the extrasynaptic plasma membrane (arrows) of interneurons (panel D) or dendritic spines (s) and shafts (Den) of pyramidal cells contacted by axon terminals (at). Few immunoparticles were observed at intracellular sites (crossed arrows) in dendritic spines and shafts, and even fewer (arrowheads) presynaptically in axon terminals (at); (**G**) Compartmentalisation of Kv4.3 in CA3 pyramidal cells and DG granule cells. Bar graphs showing the percentage of immunoparticles for Kv4.3 at post- and presynaptic compartments, and along the plasma membrane and intracellular sites in dendritic spines, dendritic shafts and axon terminals. A total of 3075 immunoparticles in the CA3 region and 2607 immunoparticles in the DG were analysed. Most immunoparticles were postsynaptic, both along the plasma membrane and at cytoplasmic sites. Postsynaptically, immunoparticles were detected in dendritic spines and in dendritic shafts; (**H**) Histogram showing the distribution of immunoreactive Kv4.3 in relation to glutamate release sites in dendritic spines of CA3 pyramidal cells and DG granule cells. Data are expressed as the proportion of immunoparticles at a given distance from the edge of the synaptic specialisation. These data show that Kv4.3 immunoparticles were distributed identically in spines of the CA3 and DG and in the proximity of asymmetrical synapses. Scale bars: **A**,**B**,**D**,**F**, 500 nm; **C**,**E**, 200 nm.

From the light microscopic analysis, it was not possible to predict whether any of the immunoreactivity was associated with a specific compartment or instead it was distributed uniformly on the neuronal surface. Therefore, to investigate the subcellular localisation of Kv4.2 and Kv4.3 with high spatial resolution in the different regions of the hippocampus, both channel subtypes were studied with pre-embedding immunogold methods combined with quantitative analyses on tissue blocks taken from the distal part of the *stratum radiatum* of the CA1 area, the *stratum radiatum* of the CA3 area and the molecular layer of the DG (Figure 7 and Figure 8).

#### 2.4.1. Subcellular localisation of Kv4.2. 

Immunoreactivity for Kv4.2 was primarily found along the extrasynaptic plasma membrane of dendritic spines and shafts of pyramidal cells and granule cells (53.81% in the CA1 region; 58.95% in the CA3 region; and 59.69% in the DG) and also associated at intracellular sites with endoplasmic reticulum cisterna in dendritic shafts and the spine apparatus in dendritic spines (46.19% in the CA1 region; 41.05% in the CA3 region; and 40.31% in the DG) (Figure 7A,C–H). Of the immunoparticles found in the plasma membrane, most (98.82% in the CA1 region; 98.86% in the CA3 region; and 98.94% in the DG) were found in postsynaptic compartments, mainly in dendritic spines of CA1 pyramidal cells (Figure 7A,H), CA3 pyramidal cells (Figure 7D,H) and granule cells of the DG (Figure 7F,H), where they were distributed along the extrasynaptic plasma membrane or at the edge of asymmetrical synapses established by excitatory axon terminals. In addition of principal cells, few Kv4.2 immunoparticles were found along the extrasynaptic plasma membrane and intracellular sites of interneurons (Figure 7B), identified by the lack of spines and convergence of synapses on their shafts. Interestingly, immunoparticles for Kv4.2 were also detected in the postsynaptic membrane specialisation of GABAergic synapses (Figure 7C,E,G), identified based on ultrastructural criteria like presynaptic clusters of synaptic vesicles and widening of the extracellular space. In the DG, the Kv4.2-immunopositive GABAergic synapses were established in the somata of granule cells (Figure 7G), indicating that the axon terminals belong to basket cells. Presynaptically, very weak immunoreactivity for Kv4.2 (1.18% in the CA1 region; 1.14% in the CA3 region; and 1.06% in the DG) was occasionally detected in axon terminals making asymmetrical synapses with dendritic spines (Figure 7B,D). Immunoparticles were localised either to the extrasynaptic plasma membrane or to the active zone of axon terminals.

To investigate the location of Kv4.2 immunoparticles relative to glutamate release sites and to compare this distribution in different hippocampal subfields, we analysed the position of immunoparticles in relation to the closest edge of the postsynaptic membrane specialisation of dendritic spines of pyramidal in the CA1 and CA3 regions, as well as DG dendritic spines (CA1, *n* = 354 immunoparticles on 93 spines; CA3, *n* = 313 immunoparticles on 93 spines; DG, *n* = 376 immunoparticles on 93 spines) (Figure 7I). The data showed that around 47% of immunoparticles in CA1, 46% in CA3 and 45% in DG were located within a distance of 300 nm from the edge of postsynaptic densities (PSDs) (Figure 7I). This analysis revealed that Kv4.2 share the same subcellular distribution and abundance in dendritic spines of CA1, CA3 and DG, and also that nearly half of the total amount of immunoparticles for Kv4.2 were close to PSDs.

#### 2.4.2. Subcellular localisation of Kv4.3. 

Immunoreactivity for Kv4.3 was detected in principal cells and interneurons in a subfield-dependent manner (Figure 8A–F). In the CA1 region, Kv4.3 was primarily found in the somata and dendritic shafts of interneurons, identified by the lack of dendritic spines and the convergence of symmetrical and asymmetrical synapses on the shaft (Figure 8A–C). Immunoparticles for Kv4.3 were mainly localised along the extrasynaptic plasma membrane of shafts and also at cytoplasmic sites associated with small cisterna or vesicles of the endoplasmic reticulum (Figure 8A–C). Immunoreactivity for Kv4.3 was frequently detected at the edge of asymmetrical synapses on dendritic shafts (Figure 8A,B). Similarly to Kv4.2, in addition to extrasynaptic membranes, immunoparticles for Kv4.3 were also observed along the postsynaptic membrane specialisation of GABAergic synapses established by axon terminals with dendritic shafts of interneurons (Figure 8C). Very few immunoparticles were detected in presynaptic axon terminals establishing synaptic contact with interneurons (Figure 8A). In the CA3 region and DG, immunoreactivity for Kv4.3 was detected in interneurons, both in plasma membrane and cytoplasmic sites (Figure 8D–F). In addition to interneurons, immunoreactivity for Kv4.3 was also detected along the extrasynaptic plasma membrane of dendritic spines and shafts of pyramidal cells and granule cells (58.90% in the CA3 region; and 59.53% in the DG) and also located at intracellular sites (41.10% in the CA3 region; and 40.46% in the DG) (Figure 8E–G). Most of the immunoparticles detected in the plasma membrane (98.28% in the CA3 region; and 98.31% in the DG) were found in postsynaptic compartments, mainly in dendritic spines (66.70% in the CA3 region; and 61.73% in the DG), where they were distributed along the extrasynaptic plasma membrane or at the edge of asymmetrical synapses (Figure 8E–G). At presynaptic sites, few immunoparticles for Kv4.3 (1.72% in the CA3 region; and 1.69% in the DG) were detected along the extrasynaptic plasma membrane or at the active zone of axon terminals establishing asymmetrical synapses with dendritic spines (Figure 8E).

To establish the relative densities of Kv4.3 in spines relative to glutamate release sites, measurements were taken from the *stratum radiatum* of the CA3 area and the molecular layer of the DG. We analysed the position of immunoparticles in relation to the closest edge of the postsynaptic membrane specialisation of dendritic spines of pyramidal cells in the CA3 region and granule cells in the DG (CA3, *n* = 371 immunoparticles on 93 spines; DG, *n* = 389 immunoparticles on 93 spines) (Figure 8H). Data was displayed as frequency of particles in arbitrarily chosen 60 nm wide segments of the membrane of dendritic spines, starting at the edge of the synaptic membrane specialisation. The data expressed in this way show the change in density of immunoparticles for Kv4 depending on the distance from the synapse. Our results showed that around 51% of all channel immunoparticles in CA3 and 53% of all channel immunoparticles in DG were located within a distance of 300 nm from the edge of PSDs (Figure 8H). This analysis revealed that Kv4.3 share the same subcellular distribution and abundance in dendritic spines of CA3 and DG, and also that Kv4.3 is enriched in an annulus of 300 nm around PSDs.

Altogether, our data show that both Kv4.2 and Kv4.3 have very similar distribution patterns in the spines of principal cells and along the surface of granule cells, and also providing evidence for their non-uniform distribution over the plasma membrane of hippocampal neurons.

### 2.5. Co-Localisation of Kv4.2 and Kv4.3 as Detected by Electron Microscopy

**Figure 9:**
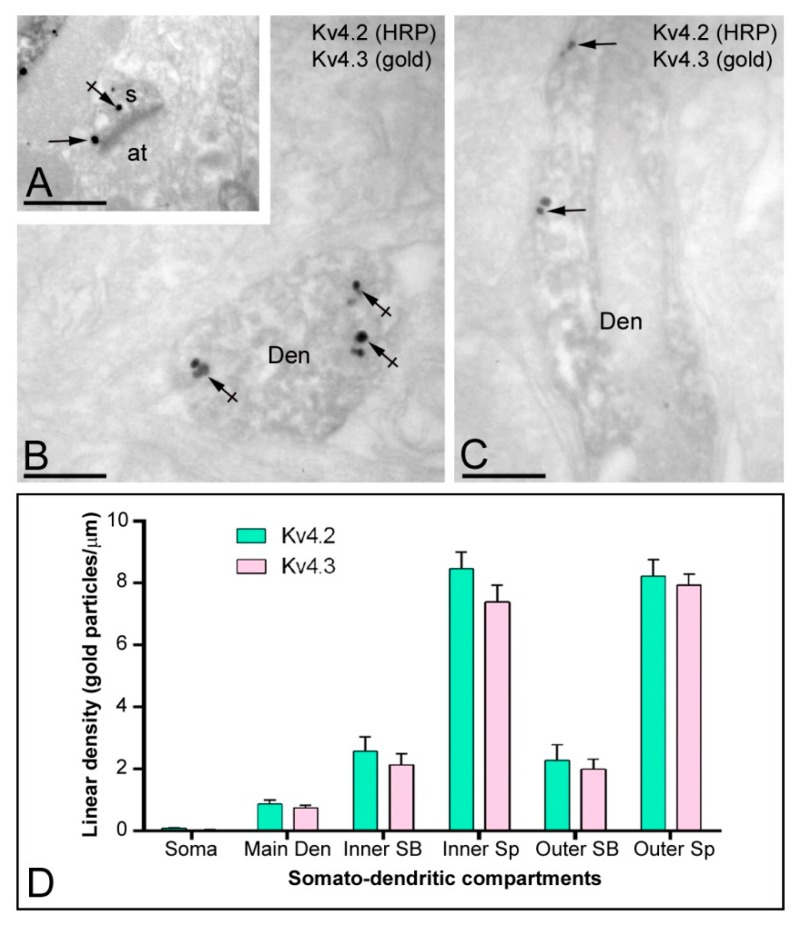
Co-localisation of Kv4.2 and Kv4.3 in granule cells. (**A**–**C**) Electron micrographs showing co-localisation for Kv4.2 with Kv4.3, as detected using a double-labelling pre-embedding immunogold method at P60. Labelling is shown with immunoperoxidase reaction for Kv4.2, and with silver-intensified immunogold reaction for Kv4.3. Immunoparticles for Kv4.3 were seen in dendritic spines (s) and shafts (Den) of granule cells immunopositive for Kv4.2, both along the plasma membrane (arrows) and at intracellular sites (crossed arrows); (**D**) Change in the density of Kv4.2 and Kv4.3 in DG granule cells as a function of distance from the soma in six somato-dendritic domains. Density of immunoparticles for the two channel subtypes increased significantly from soma to dendritic spines (Sp) in the inner one-third and outer two-thirds of the molecular layer. This analysis demonstrated their similar non-uniform distributions over the neuronal surface of granule cells. Inner SB, spiny branchlets in the inner one-third; Outer SB, spiny branchlets in the outer two-third; Inner Sp, spines in the inner one-third; Outer Sp, spines in the outer two-third. Scale bars: **A**–**C**, 500 nm.

The similar labelling pattern detected at the light microscopic level and the parallel distribution observed by electron microscopy suggests that Kv4.2 co-localise with Kv4.3 in the same neuronal compartments of granule cells. To confirm this directly, double-labelling immunohistochemistry was performed. Kv4.2 immunoreactivity was visualised by the immunoperoxidase reaction and Kv4.3 immunoreactivity was revealed with the silver-intensified immunogold reaction. Using this approach, we found extensive co-localisation of immunoreactivity for Kv4.2 channels with Kv4.3 in the same subcellular compartment of granule cells (Figure 9A–C).

We next analysed the plasma membrane distribution of Kv4.2 and Kv4.3 as a function of distance from the soma, in order to determine the abundance of the two channels along the neuronal surface of granule cells (Figure 9D). Quantification of immunogold reactions in six somato-dendritic domains of DG granule cells showed a distance-dependent increase from soma to dendritic spines in the molecular layer (Figure 9D). Thus, density was low in the somata (0.09 ± 0.01 immunoparticles/µm^2^ for Kv4.2; and 0.04 ± 0.01 immunoparticles/µm^2^ for Kv4.3), increased in the main dendrites in the molecular layer (0.87 ± 0.13 immunoparticles/µm^2^ for Kv4.2; and 0.74 ± 0.09 immunoparticles/µm^2^ for Kv4.3), increased again in oblique dendrites in the inner one-third (2.57 ± 0.46 immunoparticles/µm^2^ for Kv4.2; and 2.12 ± 0.34 immunoparticles/µm^2^ for Kv4.3) and outer two-thirds (2.28 ± 0.49 immunoparticles/µm^2^ for Kv4.2; and 1.99 ± 0.33 immunoparticles/µm^2^ for Kv4.3) of the molecular layer, and was highest in the dendritic spines in the inner one-third (8.47 ± 0.52 immunoparticles/µm^2^ for Kv4.2; and 7.39 ± 0.53 immunoparticles/µm^2^ for Kv4.3) and outer two-thirds (8.23 ± 0.54 immunoparticles/µm^2^ for Kv4.2; and 7.92 ± 0.38 immunoparticles/µm^2^ for Kv4.3) of the molecular layer (Figure 9D). This analysis demonstrated that the two channels followed non-uniform distributions over the dendritic surface of granule cells and also that the density of Kv4.2 and Kv4.3 followed a very similar pattern.

## 3. Discussion

Understanding how information is processed within and between subfields and dendritic layers of the hippocampus requires a thorough characterisation of the distribution and abundance of voltage-gated ion channels. Establishing the expression, subcellular localisation and frequency of Kv4 channels is of particular interest given the important roles of this channel subfamily in controlling neuronal excitability and synaptic plasticity [21]. To this end, our data unravelled previously uncharacterised Kv4.2 and Kv4.3 developmental expression and subcellular localisation, as discussed below.

### 3.1. Differential Expression of Kv4.2 and Kv4.3 Channels in the Adult and Developing Brain

A-type channels generate a transient outward current that exhibits rapid activation and inactivation and are of significant importance with respect to hippocampal physiology in the determination of action potential shape and the rate of spike production [6]. Among A-type channels, Kv4 currents are pharmacologically very similar and also have similar activation properties [7,22,23,24,25]. This fact favoured the idea that the different Kv4 channel subtypes were distributed in a region- and cell type-specific manner. Our histoblot data showed that Kv4.2 and Kv4.3 proteins were widely expressed throughout the brain, consistent with previous in situ hybridisation and immunohistological studies [14,15,16,17]. We demonstrated that the Kv4.2 and Kv4.3 channels were distributed in a number of brain regions where somato-dendritic A-currents have been reported using electrophysiological techniques. These regions include the hippocampus, cerebellum, cortex, caudate putamen, septum and thalamic nuclei, and in all of them A-currents are important determinants of action potential threshold, waveform, and firing frequency [6,7,26,27].

The intensity of immunolabelling for Kv4.2 and Kv4.3 channels varied markedly at the protein level between specific brain regions and showed notable regional differences in their expression like in the cerebellum, septum and thalamic nuclei. For instance, in the cerebellum strong immunoreactivity for Kv4.2 and Kv4.3 was detected in the granule cell layer and molecular layer, respectively, suggesting their segregation in different cerebellar neuron populations. In some other regions, such as the hippocampus, cortex and caudate putamen, Kv4.2 and Kv4.3 channels displayed overlapping expression. In the hippocampus, strong immunoreactivity for Kv4.2 and Kv4.3 was observed in the CA3 region and in the dentate gyrus, suggesting their co-expression in the hippocampal cells of these areas. Our data is in agreement with previous mRNA, protein and functional studies showing Kv4.2 and Kv4.3 expression in those hippocampal regions [7,14,15,16,17].

One sign of the functional importance of K^+^ channel diversity comes from examining the mRNA or protein changes during development. K^+^ channels have been involved in a number of functions during development, including neurites outgrowth, cell differentiation, neurogenesis, and synapse formation and elimination [28]. While the temporal expression of G protein-inwardly rectifying K^+^ (GIRK) channels and small conductance K^+^ (SK) channels is mostly known [20,29,30], data regarding the developmental expression of Kv4 channels are very limited and only restricted to few brain regions. In the present study, the expression of Kv4.2 and Kv4.3 was detected in the brain since the day of birth and increased throughout postnatal development, but showed changes depending on each of the two channels and the specific brain region. The developmental profile detected in the cortex for the two channels is consistent with previous electrophysiological studies showing that A-type current components increase with postnatal age [31]. In the cerebellum, the developmental changes described in Kv4.2 channel expression in granule cells correspond to the change in K^+^ currents observed using the whole cell patch clamping methods [27]. Similarly, spatio-temporal expression Kv4.3 is consistent with previous immunohistochemical studies in cerebellar neurons [32].

### 3.2. Cellular and Subcellular Localisation of Kv4.2 and Kv4.3 Channels in the Hippocampus

To understand the influence of A-type currents underlaid by Kv4 channels on synaptic processing in synaptic circuits, we need to know how they are distributed in different cell types, dendrites and at synapses of hippocampal circuits. To this end, at the light microscopic level, Kv4.2 and Kv4.3 were widely distributed in the hippocampus. Pyramidal cells of the CA3 and granule cells of the dentate gyrus express both Kv4.2 and Kv4.3 channels, while CA1 pyramidal cells only express Kv4.2, in agreement with the expression of mRNA and protein using in situ hybridisation and immunohistochemical methods, respectively [14,15,16,17,33,34]. Thus, the laminar distribution of Kv4.2 and Kv4.3 was similar in CA3 and dentate gyrus but it remains to be established whether they occur at the same sites in the plasma membrane.

In accordance with the histoblot analyses that indicated a similar intensity of Kv4.2 and Kv4.3 in the dendritic layers of CA3 and dentate gyrus, immunoelectron microscopy revealed a very similar subcellular distribution of the two channel subtypes in principal cells. In these two hippocampal subfields, excitatory inputs are provided mostly by entorhinal, commissural and local collateral afferents [1]. Kv4.2 and Kv4.3 immunoreactivity was observed mostly in postsynaptic compartments (around 99% of immunoparticles for Kv4.2 and up to 98.3% for Kv4.3), although a very low frequency of pre-synaptic labelling was also detected in axon terminals (around 1% of immunoparticles for Kv4.2 and up to 1.7% for Kv4.3). Although the two channel subtypes can be found at any position on the somato-dendritic domain, the density of Kv4.2 and Kv4.3 on spines was much higher in spines than in dendritic shafts of pyramidal and granule cells, suggesting that they are related to excitatory inputs. The quantitative comparison of dendritic spines in the different hippocampal regions revealed that around half of all immunoparticles for Kv4.2 and Kv4.3 were located within a distance of 300 nm from the edge of postsynaptic specialisation. A very similar subcellular and quantitative distribution was found for Kv4.2 in pyramidal cells of the CA1 region, whose axon terminals establishing excitatory synapses on dendritic spines mostly originate from Schaffer collateral/commissural, entorhinal and a few thalamic and local collateral afferents [1]. Altogether, the immunolabelling of dendritic spines of principal cells and the proximity to PSDs indicate that Kv4.2 and Kv4.3 could be modulated by synaptic activity. Indeed, several functional studies demonstrated that A-type currents are synaptically regulated and control action potential backpropagation into dendrites of principal cells. This activates Cav channels, Nav channels and NMDA receptors, influencing the dendritic integration of synaptic inputs [22,24,35,36]. Taking together the existing information, the most likely channel subtype underlying A-type currents in somato-dendritic domains of CA1 pyramidal cells is Kv4.2, and in CA1 interneurons is Kv4.3, but in CA3 pyramidal cells and dentate gyrus granule cells are both Kv4.2 and Kv4.3. Nevertheless, the possibility that the other channel subtypes that underlie A-type currents like Kv1.4, Kv3.4 and Kv4.1 are also involved cannot be excluded.

Other hippocampal neuron population expressing mostly Kv4.3, but also Kv4.2, are the interneurons. Thus, Kv4.3 is located in different population of interneurons distributed throughout all strata of the CA1 and CA3 regions and the dentate gyrus. Interneurons immunopositive for Kv4.3 are cholecystokinin, calbindin and somatostatin GABA-containing types [37]. The Kv4.2-immunolabelled interneurons could not be positively identified as any particular subtype in the absence of information about their axonal patterns or neurochemical characteristics, but antibodies to Kv4.2 probably label a different population from that expressing Kv4.3. Thus, Kv4 seems to be important for A-type K^+^ currents and regulation of somato-dendritic excitability in interneuron subpopulations, supporting specific roles of Kv4 channels in hippocampal rhythmic activity [37].

In addition to their distribution along the somato-dendritic compartments of principal cells and interneurons as already described, Kv4.2 and Kv4.3 are concentrated in the postsynaptic membrane specialisation, directly apposed to the presynaptic terminal. The question of whether Kv4.2 and/or Kv4.3 are present in postsynaptic specialisations of chemical synapses or in non-synaptic specialisation still remains a controversial issue. Using electron microscopic techniques, several studies reported that Kv4 channels can be located at synaptic sites in the hippocampus, supraoptic nucleus, cerebellum and visual cortex [34,38,39,40]. However, some other studies concluded that the aggregation of channels was present at sites distinct from chemical synapses in the main olfactory bulb, cerebellum and CA1 region of the hippocampus [41,42]. In the present study, we found ultrastructural evidence that Kv4.2 and Kv4.3 channels are located in GABAergic synapses in hippocampal neurons. Immunoparticles for Kv4.2 and Kv4.3 were present along the plasma membrane facing presynaptic clusters of synaptic vesicles and widening of the extracellular space, considered universal ultrastructural features of chemical synapses [43]. The functional role of these Kv4.2 and Kv4.3 channels in GABAergic synapses remains to be established but might be involved in the increase of dendritic excitation by attenuating inhibition or by amplifying excitation.

### 3.3. Co-Localisation and Non-Uniform Distribution of Kv4.2 and Kv4.3 Channels in Granule Cells

The similarity in the pattern of immunostaining for Kv4.2 and Kv4.3, observed in light microscope, suggested their co-localisation. Indeed, double-labelling immunoelectron microscopy revealed the presence of the two channels in the same granule cell and same subcellular compartments. Quantitative analyses revealed that the density of Kv4.2 and Kv4.3 immunoparticles increased significantly from the soma towards dendritic spines, demonstrating an uneven distribution of the two channel subtypes along the neuronal surface of granule cells. The same somato-dendritic gradient has been previously described in hippocampal neurons for different ion channels, including Cav3.2, GIRK and SK channels [20,29,30,44]. Uneven distributions of Kv channels have also been described in other brain areas like the cerebellum [39,41], visual cortex [40] or supraoptic nucleus [38].

The similar somato-dendritic gradient found for Kv4.2 and Kv4.3 along the neuronal surface of granule cells favours the idea that they are likely to form heterotetrameric Kv4.2/Kv4.3 channels in granule cells. Our data further suggest that granule cells use similar mechanisms to traffic and target these channels to the subcellular compartments. The molecular mechanisms that regulate the distribution of Kv4.2 and Kv4.3 proteins might involve a variety of identified proteins that interact with Kv4 channels and are also known to alter their expression, including voltage-dependent potassium channel interacting proteins (KChIPs), dipeptidyl peptidase-like proteins (DPLPs), Kvβ subunits, the acting-binding protein filamin or the scaffolding protein PSD95 [6,45].

## 4. Material and Methods

### 4.1. Animals

Four adult male Wistar rats (200–250 g; Charles River Laboratories, Barcelona, Spain) housed in the Animal House Facilities of the Universidad de Castilla-La Mancha (Albacete, Spain) were used in the present study. Care and handling of animals prior to and during experimental procedures was in accordance with European Union regulations (86/609/EC), and the protocols were approved and supervised by the local Animal Care and Use Committee(PR-2014-07-05, 19 July 2014).

For histoblotting, OF-1 mice used were from different litters and were grouped as follows: P0, P5, P10, P15, P21, P30 and P60 (*n* = 3 per group). The animals were deeply anaesthetised by intraperitoneal injection of ketamine/xylazine 1:1 (0.1 mL/kg body weight) and the brains were quickly frozen in liquid nitrogen. For immunohistochemistry at the electron microscopic level, the animals used were P30 (*n* = 3). The animals were anaesthetised by intraperitoneal injection of ketamine/xylazine 1:1 (0.1 mL/kg b.w.). Once reflex activity was completely abolished, the heart was surgically exposed for perfusion fixation through the ascending aorta, first with 0.9% saline and then followed by freshly prepared ice-cold fixative containing 4% paraformaldehyde, with 0.05% glutaraldehyde and 15% (*v*/*v*) saturated picric acid made up in 0.1 M phosphate buffer (PB, pH 7.4). After perfusion, brains were removed and immersed in the same fixative for 2 h or overnight at 4 °C. Tissue blocks were washed thoroughly in 0.1 M PB. Coronal 60 μm thick sections were cut on a Vibratome (Leica V1000, Barcelona, Spain).

### 4.2. Antibodies and Chemicals

The following primary antibodies were used: monoclonal anti-Kv4.2 (K57/1; aa. 209–225 of human Kv4.2, extracellular S1-S2 loop, Q9NZV8; NeuroMab, UC Davis/NIH, Davis, CA, USA); monoclonal anti-Kv4.3 (K75/41; aa. 415–636 of rat Kv4.3, cytoplasmic C-terminus, Q62897; NeuroMab, UC Davis/NIH, Davis, CA, USA); rabbit anti-Kv4.2 polyclonal (APC-023; Alomone Labs., Jerusalem, Israel), and guinea pig anti-Kv4.3 polyclonal (aa. 607–636 of mouse Kv4.3; GP-Af390, Frontier Institute co., Sapporo, Japan). The characteristics and specificity of the monoclonal antibodies targeting Kv4.2 and Kv4.3 has been described by the manufacturer (http://neuromab.ucdavis.edu/catalog.cfm).

The secondary antibodies used were as follows: Alkaline phosphatase (AP)-goat anti-mouse IgG (H+L) (1:5000; Sigma-Aldrich, Sant Louis, MO, USA), biotinylated goat anti-mouse IgG and biotinylated goat anti-rabbit IgG (Vector Laboratories, Burlingame, CA), and goat anti-mouse and anti-rabbit IgG coupled to 1.4 nm gold (1:100; Nanoprobes Inc., Stony Brook, NY, USA).

### 4.3. Histoblotting

The regional distribution of A-type channel subunits was analysed in rodent brains, using an in situ blotting technique (histoblot) [19]. For this technique, the expression patterns for Kv4.2 and Kv4.3 were determined in mouse brains. Briefly, horizontal cryostat sections (10 μm) from mouse brain were apposed to nitrocellulose membranes moistened with 48 mM Tris-base, 39 mM glycine, 2% (*w*/*v*) sodium dodecyl sulphate and 20% (*v*/*v*) methanol for 15 min at room temperature (~20 °C). After blocking in 5% (*w*/*v*) non-fat dry milk in phosphate-buffered saline, nitrocellulose membranes were treated with DNase I (5 U/mL), washed and incubated in 2% (*w*/*v*) sodium dodecyl sulphate and 100 mM β-mercaptoethanol in 100 mM Tris–HCl (pH 7.0) for 60 min at 45 °C to remove adhering tissue residues. After extensive washing, the blots were reacted with affinity-purified anti-Kv4.2 and anti-Kv4.3 antibodies (0.5 mg/mL) in blocking solution overnight at 4 °C. The bound primary antibodies were detected with alkaline phosphatase-conjugated anti-mouse or anti-rabbit IgG secondary antibodies. A series of primary and secondary antibody dilutions and incubation times were used to optimise the experimental conditions for the linear sensitivity range of the alkaline phosphatase reactions. To compare the expression levels of each protein during development, all nitrocellulose membranes were processed in parallel, and the same incubation time for each reagent was used for all antibodies at all ages. We only compared labelling intensities obtained with the same antibody.

Digital images were acquired by scanning the nitrocellulose membranes using a desktop scanner (HP Scanjet 8300). Image analysis and processing were performed using the Adobe Photoshop software (Adobe Systems, San Jose, CA, USA) as described previously [20]. The same incubation time was used for all reagents. All of the images were processed with the same equipment in the same way to allow comparison of the intensity of grey scale images at different postnatal ages and in different brain regions on different days. The pixel density (expressed as arbitrary units) of immunoreactivity was measured using open circular cursors with a diameter of 0.10 mm. The cursors were placed in different brain regions identified based on the adjacent cresyl violet-stained sections. We used background correction to eliminate potential differences in optical densities across different sections in different experiments. The average of eight background determinations carried out near the brain protein-containing areas of the immunostained nitrocellulose membranes was subtracted from the average pixel densities measured within brain regions. Following background corrections, the average pixel density for the whole region from one animal counted as one “*n*”. Under these conditions, labelling performed on different days produced very consistent results. Data were analysed and plotted using a software analysis (Soft Imaging Systems, Munster, Germany).

### 4.4. Immunohistochemistry for Light Microscopy

Immunohistochemical reactions at the light microscopic level were carried out using the immunoperoxidase method as described earlier [46]. Briefly, sections were incubated in 10% normal goat serum (NGS) diluted in 50 mM Tris buffer (pH 7.4) containing 0.9% NaCl (TBS), with 0.2% Triton X-100, for 1 h. Sections were incubated in anti-Kv4.2 or anti-Kv4.3 antibodies (1–2 µg/mL diluted in TBS containing 1% NGS), followed by incubation in biotinylated goat anti-rabbit IgG or anti-mouse IgG (Vector Laboratories, Burlingame, CA, USA) diluted 1:200 in TBS containing 1% NGS. Sections were then transferred into avidin–biotin–peroxidase complex (ABC kit, Vector Laboratories). Bound peroxidase enzyme activity was revealed using 3,3-diaminobenzidine tetrahydrochloride (DAB; 0.05% in TB, pH 7.4) as the chromogen and 0.01% H_2_O_2_ as the substrate. Finally, sections were air-dried and mounted prior to observation in a Leica photomicroscope (DM2000) equipped with differential interference contrast optics and a digital imaging camera.

### 4.5. Immunohistochemistry for Electron Microscopy

Immunohistochemical reactions for electron microscopy were carried out using the pre-embedding immunogold method described previously [46]. Briefly, free-floating sections were incubated in 10% (*v*/*v*) NGS diluted in TBS. Sections were then incubated in anti-Kv4.2 or anti-Kv4.3 antibodies (3–5 μg/mL diluted in TBS containing 1% (*v/v*) NGS), followed by incubation in goat anti-rabbit IgG or anti-mouse IgG coupled to 1.4 nm gold (Nanoprobes Inc., Stony Brook, NY, USA), respectively. Sections were postfixed in 1% (*v*/*v*) glutaraldehyde and washed in double-distilled water, followed by silver enhancement of the gold particles with an HQ Silver kit (Nanoprobes Inc.). In co-labelling experiments, Kv4.2 immunoreactivity was visualised by the immunoperoxidase reaction, and Kv4.3 immunoreactivity was revealed with the silver-intensified immunogold reaction. Secondary antibody mixtures included biotinylated goat anti-mouse (diluted 1:100; Vector Laboratories) and goat anti-rabbit (diluted 1:100) coupled to 1.4 nm gold (Nanoprobes), both diluted in 1% NGS/TBS. Sections from single labelling or double labelling were then treated with osmium tetraoxide (1% in 0.1 M phosphate buffer), block-stained with uranyl acetate, dehydrated in graded series of ethanol and flat-embedded on glass slides in Durcupan (Fluka) resin. Regions of interest were cut at 70–90 nm on an ultramicrotome (Reichert Ultracut E, Leica, Vienna, Austria) and collected on single slot pioloform-coated copper grids. Staining was performed on drops of 1% aqueous uranyl acetate followed by Reynolds’s lead citrate. Ultrastructural analyses were performed in a Jeol-1010 electron microscope.

### 4.6. Quantification of Kv4 Channel Immunoreactivities

To establish the relative abundance of Kv4.2 and Kv4.3 channel immunoreactivity in different compartments of pyramidal cells and granule cells, we used 60-μm-thick coronal slices processed for pre-embedding immunogold immunohistochemistry. The procedure was similar to that used previously [44]. Briefly, for each of the three adult animals, three samples of tissue were obtained for the preparation of embedding blocks, thus using in total nine blocks. To minimise false negatives, electron microscopic serial ultrathin sections were cut close to the surface of each block, as immunoreactivity decreased with depth. We estimated the quality of immunolabelling by always selecting areas with optimal gold labelling at approximately the same distance from the cutting surface. Randomly selected areas were then photographed from the selected ultrathin sections and used with final magnification between 30,000 and 50,000×. Quantification of immunogold labelling was carried out in reference areas of CA1, CA3 and DG totalling approximately 2500 µm^2^ in each hippocampal area. Quantification of immunolabelling was performed in three different ways:

Percentage of immunoparticles for Kv4 channels. To study the frequency of Kv4.2 and Kv4.3 channel immunoreactivity, we counted immunoparticles identified in each reference area (CA1, CA3 and DG) and present in different subcellular compartments: dendritic spines, dendritic shafts and axon terminals. The data were expressed as a percentage of immunoparticles in each subcellular compartment, both in the plasma membrane and at intracellular sites.

Distribution of Kv4 channels relative to glutamate release sites. To determine the relative abundance of Kv4.2 and Kv4.3 channel immunoreactivity in dendritic spines of CA1 and CA3 pyramidal cells and granule cells of the dentage gyrus, and their association with excitatory synapses, immunoparticles identified in each reference area were counted. As differences in the distribution of gold particles among samples were not statistically significant (*p* > 0.43, *Kolmogorov–Smirnov non-parametric test*), the data were pooled. We then measured the length of the dendritic spine membrane from the edge of the synaptic junction. The position of the centre of each immunoparticle attached to the plasma membrane of the dendritic spine was measured as a function of distance from the edge of the postsynaptic density using a digitising tablet and appropriate software (ImageJ, NIH, Bethesda, MD, USA). Finally, to obtain a normalised value of the relative abundance of Kv4.2 and Kv4.3 channels along the dendritic spines, the number of gold particles was expressed as relative frequency in bins corresponding to 60-nm membrane segments of spine membrane.

Density gradient of Kv4 channels along the surface of granule cells. To establish the organisation and density of Kv4.2 and Kv4.3 along the surface of granule cells in the dentate gyrus, the hippocampal region that shows strong and similar expression levels for the two channels, we performed quantification of immunolabelling in 60-µm-thick coronal slices processed for pre-embedding immunogold. Quantitative analysis of immunogold labelling for Kv4.2 and Kv4.3 in granule cells was performed on 6 different compartments: somata in the granule cell layer, main dendrite in the molecular layer, spiny branchlets (oblique dendrites) in the inner 1/3 of the molecular layer, spiny branchlets in the outer 2/3 of the molecular layer, dendritic spines in the inner 1/3 of the molecular layer and dendritic spines in the outer 2/3 of the molecular layer. Main dendrites were identified based on their large diameter and the absence of dendritic spines, while spiny branchlets were identified based on their small diameter and the presence of at least one emerging spine from the dendritic shaft. Immunoparticles identified in the plasma membrane of granule cells were counted and the area of the subcellular compartment containing the immunoparticles was measured (ImageJ). The data, linear density of Kv4.2 and Kv4.3 in each neuronal compartment, were expressed as the number of immunoparticles/µm.

### 4.7. Controls

To test method specificity in the procedures for histoblots and for electron microscopy, the primary antibodies were either omitted or replaced with 5% (*v*/*v*) normal serum of the species of the primary antibody. Under these conditions, no selective labelling was observed. When double-labelling for electron microscopy was carried out, some sections were always incubated with only one primary antibody and the full complement of secondary antibodies to test for any cross-reactivity. Other sections were incubated with two primary antibodies and one secondary antibody, followed by the full sequence of signal detection. No cross-labelling was detected that would influence the results. Finally, labelling patterns were also compared to those obtained by Calbindin (Swant, Marly, Switzerland); only the antibodies against Kv4.2 and Kv4.3 consistently labelled the plasma membrane.

### 4.8. Data Analysis

Statistical analyses for morphological data were performed using SigmaStat Pro (Jandel Scientific) and data were presented as mean ± SEM. Statistical significance was defined as *p* < 0.05, as determined using ANOVA (Kruskal-Wallis test) followed by the Dunns *post hoc* test for comparing all pairs of columns. For the electron microscopic data, statistical significance in the distribution of gold particles among samples was assessed with the Kolmogorov–Smirnov non-parametric test.
